# Prevalence of, and risk factors for, cognitive impairment in lacunar stroke

**DOI:** 10.1177/17474930211064965

**Published:** 2022-01-05

**Authors:** Laura Ohlmeier, Stefania Nannoni, Claudia Pallucca, Robin B Brown, Laurence Loubiere, Hugh S Markus, for the DNA Lacunar 2 Investigators

**Affiliations:** Stroke Research Group, Department of Clinical Neurosciences, University of Cambridge, Cambridge, UK

**Keywords:** Vascular cognitive impairment, small vessel disease, lacunar stroke, white matter hyperintensities

## Abstract

**Background:**

Small vessel disease (SVD) is associated with vascular cognitive impairment (VCI) but why VCI occurs in some, but not other patients, is uncertain. We determined the prevalence of, and risk factors for, VCI in a large cohort of patients with lacunar stroke.

**Methods:**

Participants with magnetic resonance imaging (MRI)-confirmed lacunar stroke were recruited in the multicenter DNA Lacunar 2 study and compared with healthy controls. A logistic regression model was used to determine which vascular risk factors and MRI parameters were independent predictors of VCI, assessed using the Brief Memory and Executive Test (BMET).

**Results:**

A total of 912 lacunar stroke patients and 425 controls were included, with mean (*SD*) age of 64.6 (12.26) and 64.7 (12.29) years, respectively. VCI was detected in 38.8% lacunar patients and 13.4% controls. In a logistic regression model, diabetes mellitus (odds ratio (OR) = 1.98 (95% confidence interval (CI) = 1.40–2.80), *p* < 0.001) and higher body mass index (BMI) (OR = 1.03 (95% CI = 1.00–1.05), *p* = 0.029) were independently associated with increased risk of VCI, and years of full-time education with lower risk (OR = 0.92 (95% CI = 0.86–0.99), *p* = 0.018). When entering both lacune count and white matter hyperintensity (WMH) in the same logistic regression model, only WMH grade was significantly associated with VCI (OR = 1.46 (95% CI = 1.24–1.72), *p* < 0.001).

**Conclusion:**

VCI is common in lacunar stroke patients, affecting almost 40%. This prevalence suggests that it should be routinely screened for in clinical practice. Risk factors for VCI in patients with lacunar stroke include diabetes mellitus, depressive symptoms, higher BMI, and WMH severity, while education is protective.

## Introduction

Lacunar stroke, usually caused by cerebral small vessel disease (SVD), accounts for a quarter of all ischemic strokes. SVD is characterized radiologically by lacunar infarcts, white matter hyperintensities (WMHs), cerebral microbleeds (CMBs), and enlarged perivascular spaces.^1^ SVD is the most common pathology underlying vascular cognitive impairment (VCI) and vascular dementia.^1^

VCI is characterized by executive dysfunction and slowing of information processing speed while episodic memory and orientation in space, time, and person are relatively preserved.^2^ Simple cognitive screening batteries commonly used in clinical practice, such as the Mini-Mental State Examination (MMSE), focus primarily on deficits in orientation and episodic memory, and are less sensitive to the cognitive profile in SVD.^2,3^ When tests more sensitive to executive function and processing speed are used, such as the Brief Memory and Executive Test (BMET), a higher prevalence of cognitive impairment is detected in patients with SVD.^2^

Cardiovascular risk factors, particularly hypertension and diabetes, increase the risk of stroke^4^ and are associated with post-stroke dementia and VCI.^5^ However, most studies include all stroke subtypes, and less data are available specifically on lacunar stroke. Apathy, a decline in goal-directed behavior, which is common in SVD, has been associated with both the degree of white matter damage and dementia in SVD.^6^ Previous studies have reported associations between VCI and the range and severity of magnetic resonance imaging (MRI) markers, such as WMH, lacunar infarcts, and CMB.^7^ Furthermore, the pathology underlying SVD may be heterogeneous and it has been suggested that there are two major pathological subtypes^8^: focal atheroma resulting in larger isolated lacunar infarcts (ILIs) and more diffuse arterial disease associated with multiple smaller lacunar infarcts. More recent studies suggest that a similar distinction can be made on MRI with patients with single lacunar infarcts, those with multiple lacunar infarcts (MLI) and with confluent WMH having distinct risk factor profiles.^9^

Most previous studies have been small, and many have used computed tomography (CT)-based phenotyping, which is less accurate in the diagnosis of lacunar stroke. Furthermore, cognitive tests sensitive to the deficit seen in SVD were not always used. In addition, whether the cognitive profile differs for the different subtypes of VCI is uncertain.

In a large prospective multicenter cohort of almost 1000 patients with MRI-confirmed lacunar stroke, we determined the prevalence of VCI measured using the BMET. We further determined risk factors associated with VCI, including cardiovascular and lifestyle risk factors, as well as MRI features.

## Methods and materials

### Study population

A case control design was used to compare patients with lacunar stroke with healthy controls. Subjects with MRI-confirmed lacunar stroke were recruited as part of the ongoing DNA Lacunar 2 study, prospectively recruiting patients from 54 hospitals across the United Kingdom. Between August 2016 and February 2021, 995 confirmed eligible patients were recruited. Ninety-one percent (*N* = 912) of participants completed the BMET and were included in the analysis.

Inclusion criteria were a clinical lacunar stroke syndrome with an anatomically corresponding lacunar infarct confirmed on MRI. Exclusion criteria were: age < 18 years; any potential cause of stroke other than SVD, including large artery stenosis > 50%, or cardio-embolic source; subcortical infarct > 15 mm diameter; any other potential cause of white matter disease, such as multiple sclerosis. Participants were recruited within 2 years of occurrence of the stroke and with an MRI performed within 1 year of index stroke. All subjects had imaging to exclude extracranial large artery stenosis.

Hypertension was defined as a systolic blood pressure > 140 mm Hg and/or diastolic blood pressure > 90 mm Hg, or treatment on antihypertensive drugs. Hyperlipidemia was defined as serum cholesterol level > 5.2 mmol/l, or treatment with lipid lowering medication. Diabetes mellitus included types 1 and 2.

Four hundred twenty-five healthy participants used as the control group were recruited from local general practitioners (GPs) or volunteer groups, as described previously.^2^ Individuals with a history of stroke, transient ischemic attack, or major neurological or psychiatric disease were excluded; however, participants with cardiovascular risk factors were included.^2^

### Review of MRI

All original MRI scans, which were performed clinically on a variety of scanners at each of the 54 recruiting sites, were reviewed with clinical details by a neurologist to confirm eligibility. All MRI scans included diffusion-weighted imaging (DWI) sequences to identify an acute infarct, fluid-attenuated inversion recovery (FLAIR), and/or T1 sequences to count old lacunar infarcts, and FLAIR and/or T2 sequences to identify WMH. Susceptibility-weighted imaging or gradient echo sequences were not mandatory, but, if available, were used to count microbleeds. The Fazekas scale^10^ was used to grade the extent of WMH on T2-FLAIR images, determined overall and separately for periventricular (PVL) and deep white matter (DWM). WMHs were graded by one of three neurologists, who underwent training and passed a test by assessment of a standardized data set prior to reading scans. Inter-observer reproducibility was assessed between raters 2 and 3, and the first rater on 30 randomly selected scans. Cohen’s kappa inter-rater reliability test showed good to very substantial agreement (rater 2: κ = 0.79 for deep WMH, κ = 0.736 for PVL-WMH, and κ = 0.793 for total WMH; rater 3: κ = 0.755 for deep WMH, κ = 0.861 for PVL-WMH, and κ = 0.782 for total WMH).

Lacunes were counted on FLAIR images, with a lacune defined as a round or ovoid subcortical fluid-filled cavity of 3–15 mm in diameter,^11^ or as an acute lacunar infarct on DWI. Lacunar stroke cases were subclassified into those with either ILI (a single lacunar infarction without confluent WMH), MLI without confluent WMH (MLI), or confluent WMH (moderate or severe confluent WMH with one or more lacunar infarcts).

### Psychological and neuropsychological assessment

Cognition was assessed using the BMET, a freely available and previously validated screening test sensitive to cognitive impairment in patients with VCI (www.BMET.info). Each sub-task is scored on an age normative scale from 0 to 2, which is used to form a total score (ranged on a scale from 0 to 16). We used the previously defined score of ⩽ 13 to define VCI.^2^ Two sub-scores, Executive Functioning and Processing Speed (EF-PS), and Orientation and Memory (OM), referring to orientation in space, time, and person, and episodic memory, were calculated, each with a score range of 0–8. The sub-score EF-PS is calculated from the sub-tasks (1) letter-number matching, (2) motor sequencing, (3) letter sequencing, and (4) number-letter sequencing, while the sub-score for OM comprised (1) orientation, (2) five-item repetition, (3) five-item recall, and (4) five-item recognition memory. As the scores are not normed for sex and education, we controlled for these variables in analysis.

The internal consistency in the SVD and the control group is adequate for the Total score and the EF-PS sub-score. The internal consistency for the OM sub-score was significantly lower (SVD, α = 0.35 and control, α = 0.45). Test–retest reliability in the SVD group was adequate for the total score, EF-PS, and OM.

The Geriatric Depression Scale (GDS), a 30-item screening questionnaire with binary yes/no answer options,^12^ was administered in the lacunar stroke participants. The GDS was divided into the two subscales, assessing depression and apathy.^13^ When testing the association between GDS depression and VCI, the item “Do you feel you have more problems with memory than most” was excluded.

### Statistical analysis

Differences in variables between lacunar stroke patients and controls, and within the different lacunar stroke subgroups, were compared using independent samples *t*-tests and chi-square tests as appropriate.

Performance on the BMET was assessed using *Z*-scores. *Z*-scores for the BMET total and sub-scores were created for lacunar stroke patients, and each lacunar stroke sub-type, and displayed in reference to healthy controls. Chi-square tests were used to compare the proportion of participants with VCI (BMET total score ⩽ 13) between groups.

Logistic regression was used to determine which vascular risk factors and MRI parameters were independent predictors of VCI. All variables were entered at the same time, as opposed to step wise.

All analyses were conducted in IBM SPSS Statistics 26.

### Standard protocol approvals, registrations, and patient consents

All participants provided signed informed consent. DNA Lacunar 2 was approved by East of England Research Ethics Committee (16/EE/0201), and control collection by London Bridge Research Ethics Committee (11/LO/0636).

## Results

### Participant characteristics

Mean age was 64.6 (*SD* = 12.26) years in the lacunar stroke cases and 64.65 (12.29) in the controls. Descriptive statistics are presented in Table 1. Within the lacunar stroke group, the mean (*SD*) number of lacunes was 2.11 (1.94), and mean WMH score was 1.31 (0.99). Ninety-five percent of participants showed an acute lesion on DWI. The median (25,75 quartile) time between index stroke and MRI was 2 days.^1,4^ The number of lacunar stroke patients in each subgroup was: ILI (360, 39.5%), MLI (126, 13.8%), and confluent WMH (426, 46.7%).

**Table 1.** Descriptive statistics for controls and lacunar patients that completed the BMET.

	Control	Lacunar (total)	ILI	MLI	Confluent WMH
*n* (%)	425	912	360 (39.5%)	126 (13.8%)	426 (46.7%)
Age, years (*SD*)	64.7 (12.29)	64.6 (12.26)	60.51 (11.85)	59.42 (11.59)	69.54 (10.83)
Sex (% female)	209 (49.2%)	338 (37.1%)	134 (37.2%)	33 (26.2%)	171 (40.1%)
mRS at consent		1.15 (1.07)	1.03 (0.96)	1.10 (1.02)	1.26 (1.17)
FTH education in years (*SD*)	1.55 (2.02)	1.28 (2.21)	1.48 (2.41)	1.27 (2.36)	1.12 (1.95)
Hypertension		665 (72.9%)	227 (63.1%)	97 (77.0%)	341 (80.0%)
Hyperlipidemia		600 (65.8%)	226 (62.8%)	80 (63.5%)	284 (69.0%)
Diabetes mellitus		189 (20.7%)	68 (18.9%)	35 (27.8%)	86 (20.2%)
Current smoker		174 (19.1%)	70 (19.4%)	30 (23.8%)	74 (17.4%)
Weekly alcohol units (*SD*)		9.86 (19.75)	10.09 (15.45)	11.91 (19.61)	9.07 (22.79)
Previous stroke		102 (11.2%)	12 (3.3%)	24 (19.0%)	66 (15.5%)
BMI kg/m^2^ (*SD*)		28.19 (6.23)	28.61 (6.02)	29.18 (5.84)	27.53 (6.44)
Lacune count (*SD*)		2.11 (1.94)	1.00 (0.00)	3.33 (1.86)	2.62 (2.24)
PVL score (*SD*)		1.24 (1.03)	0.39 (0.54)	0.79 (0.56)	2.11 (0.71)
DWM score (*SD*)		1.20 (0.96)	0.42 (0.53)	0.77 (0.54)	1.98 (0.70)
WMH score (*SD*)		1.31 (0.99)	0.45 (0.52)	0.87 (0.51)	2.17 (0.61)
GDS total (*SD*)		7.81 (6.32)	7.58 (6.53)	7.96 (6.86)	7.96 (5.99)
GDS apathy (*SD*)		2.55 (1.81)	2.37 (1.80)	2.49 (1.77)	2.71 (1.83)
GDS depression (*SD*)		5.27 (5.10)	5.21 (5.19)	5.49 (5.63)	5.25 (4.86)
VCI (%)	57 (13.4%)	354 (38.8%)	103 (28.6%)	52 (41.3%)	199 (46.7%)

BMET: Brief Memory and Executive Test; ILI: isolated lacunar infarct; MLI: multiple lacunar infarcts; WMH: white matter hyperintensity; *SD*: standard deviation; mRS: modified Rankin score^14^; FTH: full-time higher; BMI: body mass index; PVL: periventricular; DWM: deep white matter; GDS: Geriatric Depression Scale; VCI: vascular cognitive impairment.

### Cognitive data

Of the 995 lacunar stroke patients, 912 completed the BMET with a median (25,75 quartile) time between index stroke and BMET completion of 37 (5,108) days. Those who completed the BMET were younger, mean 64.6 (12.26) versus 71.7 (11.59) years (*p* < 0.001), and had less severe WMH, mean 1.31 (0.99) versus 1.81 (0.97) (*p* < 0.001).

VCI, defined as a BMET score of ⩽ 13, was present in 38.8% of lacunar cases and 13.4% of controls (*p* < 0.0001). There was a significant difference in the prevalence of VCI between the three lacunar stroke subtypes (*p* < 0.0001): 47% in confluent WMH, 41% in MLI, and 29% with ILI (Table 2).

**Table 2.** Mean and *SD* of BMET scores for controls and lacunar patients.

			Lacunar stroke subtype		
	Control	Lacunar (total)	ILI	MLI	Confluent WMH
*n*	425	912	360 (39.5%)	126 (13.8%)	426 (46.7%)
BMET total score (*SD*)	15.12 (1.54)	13.32 (3.17)	14.07 (2.51)	13.25 (3.04)	12.70 (3.56)
BMET orientation/memory (*SD*)	7.35 (1.15)	6.57 (1.86)	6.93 (1.55)	6.60 (1.86)	6.27 (2.04)
BMET executive function (*SD*)	7.77 (0.78)	6.74 (1.93)	7.14 (1.53)	6.65 (2.03)	6.42 (2.14)
Orientation (*SD*)	1.91 (0.31)	1.76 (0.53)	1.84 (0.43)	1.80 (0.49)	1.68 (0.61)
Five-item repetition (*SD*)	1.85 (0.44)	1.57 (0.74)	1.68 (0.65)	1.54 (0.77)	1.48 (0.79)
Letter–number matching (*SD*)	1.90 (0.32)	1.67 (0.59)	1.77 (0.52)	1.62 (0.66)	1.59 (0.62)
Motor sequencing (*SD*)	1.98 (0.17)	1.74 (0.61)	1.78 (0.55)	1.66 (0.67)	1.73 (0.63)
Letter sequencing (*SD*)	1.94 (0.31)	1.63 (0.71)	1.77 (0.57)	1.69 (0.64)	1.50 (0.81)
Number–letter sequencing (*SD*)	1.95 (0.28)	1.70 (0.63)	1.83 (0.51)	1.68 (0.64)	1.61 (0.70)
Delayed recall (*SD*)	1.78 (0.57)	1.63 (0.73)	1.70 (0.66)	1.64 (0.73)	1.56 (0.77)
Delayed recognition (*SD*)	1.81 (0.50)	1.62 (0.73)	1.71 (0.66)	1.62 (0.74)	1.55 (0.78)

*SD*: standard deviation; BMET: Brief Memory and Executive Test; ILI: isolated lacunar infarct; MLI: multiple lacunar infarcts; and WMH: white matter hyperintensity.

**Figure 1.**
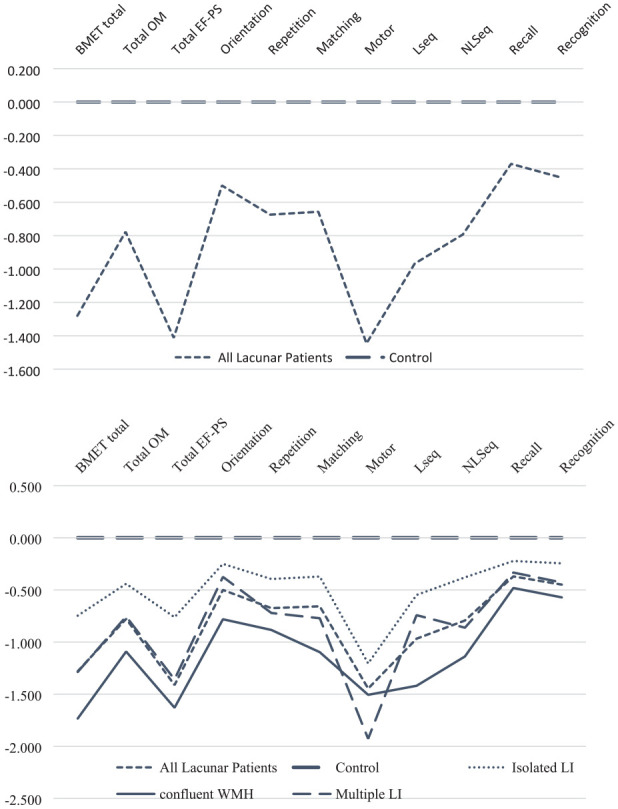
*Z*-score plot of BMET scores divided by controls and all lacunar patients and by controls and stroke subtype. Total OM: sub-score orientation and memory; total EF-PS: sub-score executive functioning and processing speed; orientation: sub-task orientation; repetition: sub-task five-item repetition; matching: sub-task letter–number matching; motor: sub-task motor sequencing; Lseq: sub-task letter sequencing; NLSeq: sub-task number–letter sequencing; recall: sub-task five-item recall; recognition: sub-task five-item recognition memory.

The profile of the cognitive impairment across sub-scores and sub-tasks is shown in the *Z*-score plot in Figure 1. Although a reduction in both the EF-PS and OM sub-scores was seen, the degree of impairment was greater for EF-PS, while episodic memory (recall) was relatively preserved. Comparing within-subject differences between the *Z*-scores, lacunar patients scored significantly lower on EF-PS sub-score compared to the OM sub-score (*p* < 0.001).

Across the lacunar stroke subtypes a similar pattern of deficits was seen (Figure 1). Cognitive performance was best in the ILI group, and worst in the confluent WMH group across all scores with the exception of motor performance where MLI performed worst.

Across all BMET sub-tasks, there was a significant difference between the control group and all lacunar patients, and the control group and each lacunar stroke subtype, apart from the sub-task Delayed Recall in the stroke subtype isolated lesion (*p* < 0.05 for all groups and sub-tasks apart from Delayed Recall in the stroke subtype isolated lesion).

### Clinical and MRI variables associated with VCI

In a logistic regression model controlling for age, sex, and time between stroke and BMET assessment, diabetes mellitus (odds ratio (OR) = 1.98 (95% confidence interval (CI) = 1.40–2.80), *p* < 0.001) and higher body mass index (BMI; OR = 1.03 (95% CI = 1.00–1.05), *p* = 0.029) were independently associated with increased risk of VCI, and years of full-time education with lower risk (OR = 0.92 (95% CI = 0.86–0.99), *p* = 0.018; Table 3).

**Table 3.** Logistic regression for the association between lifestyle and clinical variables, and VCI in lacunar patients.

				95% CI: for Exp (B)	
	Wald	Sig.	OR	Lower	Upper
Sex (female)	2.918	0.088	0.772	0.573	1.039
Age (years)	2.145	0.143	1.009	0.997	1.022
Hypertension (yes/no)	0.338	0.561	0.909	0.659	1.254
Hyperlipidemia (yes/no)	2.038	0.153	1.245	0.922	1.681
Diabetes mellitus (yes/no)	14.729	0.000	1.975	1.395	2.796
Current smoker (yes/no)	0.797	0.372	1.183	0.818	1.711
Weekly units of alcohol (per unit)	0.165	0.684	0.998	0.991	1.006
BMI (kg/m^2^)	4.774	0.029	1.027	1.003	1.053
FTH education (years)	5.635	0.018	0.921	0.861	0.986
Time between stroke and BMET	2.417	0.120	0.999	0.998	1.000

VCI: vascular cognitive impairment; OR: odds ratio; CI: confidence interval; BMI: body mass index; FTH: full-time higher; and BMET: Brief Memory and Executive Test.

When tested individually in models controlling for age, sex, education, and time between stroke and BMET assessment, both the GDS apathy score and the GDS depression score were positively associated with VCI (OR = 1.17 (95% CI = 1.08–1.26), *p* < 0.001 and OR = 1.09 (95% CI = 1.05–1.12), *p* < 0.001, respectively). However, when entering the apathy score and the depression score in the same model, only the depression score remained significantly associated with VCI (OR = 1.08 (95% CI = 1.04–1.11), *p* < 0.001).

Next, we determined the relationship between MRI markers of SVD and VCI. When tested individually in binary logistic regression models controlling for age, sex, education, and time between stroke and BMET assessment, both lacune count and WMH were associated with VCI (OR = 1.12 (95% CI = 1.05–1.20), *p* = 0.001 and OR = 1.53 (95% CI = 1.31–1.78), *p* < 0.001, respectively). However, when entering both lacune count and WMH in the same model, only WMH remained significantly associated (Table 4).

**Table 4.** Logistic regression for the association between lacune count, WMH and VCI in lacunar patients.

				95% CI: for Exp (B)	
	Wald	Sig.	OR	Lower	Upper
Lacune count	1.880	0.170	1.054	0.978	1.137
WMH score	19.906	0.000	1.458	1.235	1.720
Sex (female)	4.959	0.026	0.716	0.534	0.961
Age in years	1.777	0.183	0.992	0.979	1.004
Time between BMET and stroke (days)	1.761	0.185	0.999	0.998	1.000
FTH education (years)	6.525	0.011	0.912	0.849	0.979

WMH: white matter hyperintensity; VCI: vascular cognitive impairment; OR: odds ratio; CI: confidence interval; BMET: Brief Memory and Executive Test; and FTH: full-time higher.

When tested individually in models controlling for age, sex, education, and time between stroke and BMET assessment, both DWM-WMH and PVL-WMH were positively associated with VCI (OR = 1.51 (95% CI = 1.29–1.76), *p* < 0.0001 and OR = 1.46 (95% CI = 1.26–1.70), *p* < 0.0001, respectively). However, when entering both lacune count and WMH in the same model, only DWM-WMH remained associated (OR = 1.30 (95% CI = 1.04–1.63), *p* = 0.023).

## Discussion

In this large cohort of over 900 patients with MRI-confirmed lacunar stroke, VCI was identified in 39%. This emphasizes the high prevalence of VCI in patients with lacunar stoke, and the need for routine screening in clinical practice.

The pattern of VCI observed is consistent with previous reports of predominant deficits in executive functioning and processing speed, but relative preservation of orientation and episodic memory.^1,2^ This emphasizes the need to use tests sensitive to these domains, such as the BMET.

The size of our cohort allowed us to examine associations between cardiovascular risk factors and VCI. Diabetes mellitus, increased depressive symptoms, and higher BMI were associated with increased risk of VCI, while years of full-time education with lower risk. An association between cognitive performance and diabetes has been identified in previous research studies,^15^ suggesting that diabetic control may be important in preventing VCI. Previous smaller studies have shown associations between VCI and hyperlipidemia, alcohol consumption, and smoking, but we were unable to replicate these findings.^4^

We found a positive association with the GDS depression score and VCI. Depression has been found to be a risk factor for Alzheimer’s disease^16^ and our findings are consistent with a similar relationship for VCI. This is in alignment with previous findings that show that depressive symptoms and late life depression are associated with greater severity of WMH.^17,18^ We did not find an independent association with the apathy score and VCI. This is in contrast to previous studies that found that apathy but not depression was associated with cognition, white matter ultrastructural damage and dementia risk in SVD.^6^

Although both lacunar infarct count and WMH were associated with VCI after controlling for age and sex, when both were entered into the model only WMH remained significant. This suggests that diffuse white matter damage is the major pathology underlying VCI and dementia in this population. This is consistent with the hypothesis that diffuse white matter damage results in white matter tract disruption and disconnection of complex distributed networks relying on white matter integrity and underlying cognitive functions, such as executive functioning.^19^ There has been considerable debate as to whether WMH in different locations have different associations with cognitive impairment.^20^ We found that DWM-WMH was associated with VCI, but PVL-WMH was not, when both were entered into the same model. VCI was most common in patients with confluent WMH, intermediate in patients with MLI without WMH, and least frequent in patients with single lacunar infarcts without WMH.

Our study has a number of strengths. It prospectively recruited almost 1000 patients with MRI-confirmed lacunar stroke, which improves sensitivity of diagnosis compared with CT. We used a cognitive screening test, which has been developed and validated for the cognitive impairment in SVD.^2^

However, it also has limitations. While the BMET is a suitable test for detection of VCI, it requires participants to follow verbal instructions and complete tasks with pen and paper. In 9% of participants, the BMET was not performed, either because the participant could not complete the test or because they were unwilling to have cognitive testing. Participants who were unable to complete the BMET were older with more severe WMH, which could lead to under-reporting of the prevalence of VCI in lacunar stroke. MRI was performed as part of clinical care on a variety of different scanners and we do not have individual scanner details.

As the study design was cross-sectional, we do not know premorbid cognitive performance. Decreased cognitive performance is a comorbidity of diabetes^15^ and our study design does not allow us to determine whether patients with diabetes had lower cognitive performance prior to stroke, or whether it increases VCI risk in patients who have already developed SVD. It is likely that the VCI represents the consequences of chronic changes of SVD in addition to the effect of the lacunar stroke, particularly in those with confluent WMH.

In conclusion, our study demonstrates that VCI is common in lacunar stroke, and suggests lacunar stroke patients should be tested with an appropriate cognitive screening tool, such as the BMET. It shows that both diabetes mellitus and BMI are associated with an increased risk of VCI in patients with lacunar stroke emphasizing the need for active control of these risk factors.
